# Evaluation of photosynthetic efficacy and CO_2_ removal of microalgae grown in an enriched bicarbonate medium

**DOI:** 10.1007/s13205-015-0314-5

**Published:** 2016-01-05

**Authors:** S. Abinandan, S. Shanthakumar

**Affiliations:** Environmental Engineering Division, School of Mechanical and Building Sciences, VIT University, Vellore, 632014 India

**Keywords:** Carbon concentrating mechanism, Mixotrophic condition, *Chlorella pyrenoidosa*, Dissolved inorganic carbon, Response surface methodology

## Abstract

Bicarbonate species in the aqueous phase is the primary source for CO_2_ for the growth of microalgae. The potential of carbon dioxide (CO_2_) fixation by *Chlorella pyrenoidosa* in enriched bicarbonate medium was evaluated. In the present study, effects of parameters such as pH, sodium bicarbonate concentration and inoculum size were assessed for the removal of CO_2_ by *C. pyrenoidosa* under mixotrophic condition. Central composite design tool from response surface methodology was used to validate statistical methods in order to study the influence of these parameters. The obtained results reveal that the maximum removal of CO_2_ was attained at pH 8 with sodium bicarbonate concentration of 3.33 g/l, and inoculum size of 30 %. The experimental results were statistically significant with *R* ^2^ value of 0.9527 and 0.960 for CO_2_ removal and accumulation of chlorophyll content, respectively. Among the various interactions, interactive effects between the parameters pH and inoculum size was statistically significant (*P* < 0.05) for CO_2_ removal and chlorophyll accumulation. Based on the studies, the application of *C. pyrenoidosa* as a potential source for carbon dioxide removal at alkaline pH from bicarbonate source is highlighted.

## Introduction

Rapid exploitation of fossil fuels such as coal, petroleum, etc., has invariably increased the concentration of CO_2_ in the atmosphere to ~400 ppm (Rahaman et al. 2011). Several steps have been taken to control the CO_2_ emission rates caused during the pre and post-process of various man-made activities. Some of the process includes application of alkanolamine absorbents (Choi et al. 2012; Kim et al. 2013; Pires et al. 2011), desiccant absorption (Stewart and Hessami 2005), adsorption by activated carbon (Lu et al. 2008), mineral carbonate (Wang et al. 2008), zeolite (Wang et al. 2011), molecular sieve, ocean storage (Metz et al. 2005) and geological storage (Holloway 2007). Biofixation of CO_2_ by microalgae attracted researchers due to its affirmative behavior such as carbon neutral and biomass productivity for its various applications. However, the factors such as economic aspects, life cycle analysis, capital investment and other environment aspects have made these technological impacts slower at commercial level. Unlike other gases, CO_2_ has some significant limiting factors such as low mass transfer rate, high cost of CO_2_ capture, its transportation. Meanwhile, when the CO_2(g)_ is passed onto the aqueous solution, it reduces the pH due to the formation of H_2_CO_3_ (free CO_2_) and escapes into the atmosphere thereby resulting in significant CO_2_ loss during algae culture (Chi et al. 2011). Furthermore, pH is the major determinant of the relative concentrations of the carbonaceous system species in water and could affect the availability of carbon for algal photosynthesis in intensive cultures and at certain situations where the supply of adequate CO_2(g)_ is limited, inorganic carbon sources such as bicarbonate salts (NaHCO_3_) is best alternative to cultivate microalgae (Hsueh et al. 2009; Lam and Lee 2013; Benemann 1993). Meanwhile, it is also envisaged that mixotrophic mode of cultivating microalgae is best in terms of growth and other applications (Abreu et al. 2012; Bhatnagar et al. 2011; Cheirsilp and Torpee 2012). Based on literature, it can be established that *Chlorella* sp. is found to be most prominent and efficient in bio-fixing HCO_3_^− ^and can grew well at mixotrophic conditions (Jeong et al. 2003; Lam et al. 2012).

Accordingly, this study has attempted to add to the current knowledge for carbon sequestration by optimizing process parameters for maximum CO_2_ removal using microalgae in enriched bicarbonate medium. In this context, we aim to discuss the effect of pH, sodium bicarbonate concentration and inoculum size in mixotrophic mode of cultivation for CO_2_ removal in microalgae. In addition, we have also monitored chlorophyll as indicator for growth and photosynthetic productivity. Also, in order to optimize the chosen factors and its interactions, central composite design (CCD) using response surface methodology has been employed. The application of response surface methodology is to maximize the effective parameters and minimize the experiments as well as to study the variables individual and interactive effects (Wang et al. 2007; Zhang et al. 2009; Khataee and Dehghan 2011).

## Materials and methods

### Culturing of microalgae

*Chlorella pyrenoidosa* (NCIM 2738) was obtained from the National Centre of Industrial Microorganism (NCIM), Pune, India and was maintained in BG-11 medium (UTEX 2009) under axenic conditions. The stock solution was prepared for all the constituents in media with the following composition for 100 ml: K_2_HPO_4_ 0.4 g, CaCl_2_·2H_2_O 0.36 g, MgSO_4_·7H_2_O 0.75 g, NaNO_3_ 15 g, citric acid 0.06 g, Na_2_EDTA·2H_2_O 0.01 g, sodium carbonate 0.2 g, ammonium ferric citrate 0.06 g, and A_5_ trace solution (g/l) components as H_3_BO_3_ 2.86, MnCl_2_ 1.81, ZnSO_4_·7H_2_O 0.222, Na_2_MoO_4_·2H_2_O 0.390, CuSO_4_·5H_2_O 0.079, Co(NO_3_)_2_·6H_2_O 0.0494. From the stock solution the media has been prepared for 500 ml with 5 ml from each constituent and 0.5 ml of A_5_ trace solution. The culture was subjected to continuous illumination (14 WTL5 tungsten filament lamps; Philips Co.,) with 1500 Lux measured using TES light meter (TES CORP) at room temperature 28 ± 1 °C.

### Design of experiments

The exponential phase microalgae cells (28 × 10^5^ cells/ml)were taken for experimental studies in synthetic medium (Feng et al. 2011) with the following composition (g/l) glucose 0.4125, NH_4_Cl0.078, KH_2_PO_4_ 0.018, MgSO_4_·7H_2_O 0.013, CaCl_2_·2H_2_O 0.043, FeSO_4_·7H_2_O 0.005, A_5_ Trace solution (1 ml/l), respectively. All the experiments in the study were carried in 500 ml conical flasks containing 300 ml of working solution of synthetic medium with variables (pH, inoculum size and sodium bicarbonate). For analysis, 10 ml of sample was collected using autoclaved syringe (20 ml) for every 3 days once and was subjected for centrifugation at 8000 RPM for 10 min. The pellet was taken for estimation of chlorophyll and supernatant for CO_2_ analysis. The pH values were chosen to study the microalgae behavior to CO_2_ in acidic (pH 4 to mimic more availability of free CO_2_), pH 6 (as standard growth medium range) and alkaline range (pH 8). The sodium bicarbonate concentrations were fixed of 1–3 g per 300 ml each which was scaled up in g/l as depicted in Table 1. The inoculum sizes were fixed (10–30 %) on volume per volume basis as to envisage its effects for CO_2_ removal and the optical density of 10, 20 and 30 % inoculum sizes used in the study were recorded as 0.594, 0.692 and 0.802, respectively, measured at 660 nm using UV Visible Spectrophotometer (Cyberlab, USA). The pH of the medium was adjusted using 0.1 N NaOH and 0.1 N HCl. All the flasks were manually shaken thrice a day in order to avoid sticking of culture to flasks. The experiments were carried out in duplicates and the average has been taken RSM analysis.

**Table 1:** Experimental range and levels of independent variables

Independent variables	Design variables	Range and levels		
		−1	0	1
pH	*A*	4	6	8
NaHCO_3_ (g/l)	*B*	3.33	6.66	10
Inoculum size (%)	*C*	10	20	30

### Response surface methodology

**Fig. 1:**
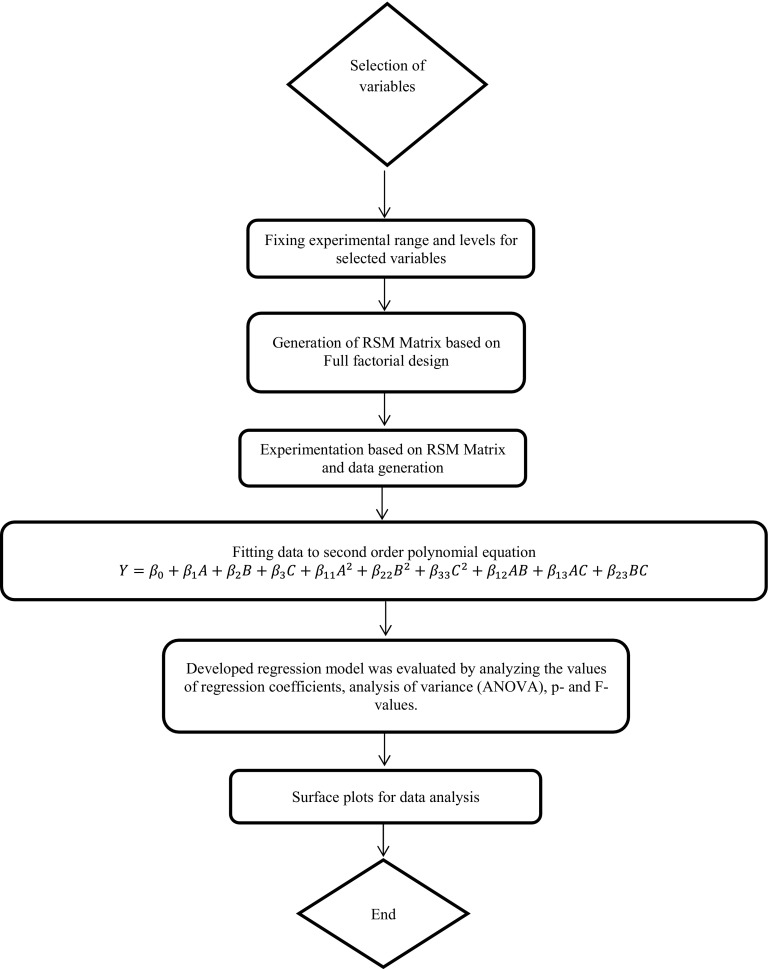
Flow chart representation of response surface methodology

In order to study the combined effects of the variables (pH, inoculum size and sodium bicarbonate) on the maximum removal of CO_2_ (%), 20 sets of experiments with appropriate combinations of pH, inoculum size and NaHCO_3_ concentration were conducted using response surface method (statistical analysis) and the details are presented in Table 2. The CCD under the response surface methodology (RSM) was employed in order to illustrate the nature of the response surface in the experimental design and to elucidate the optimal conditions of the most significant independent variables. A flowchart representation in support of the response surface methodology has been presented in Fig. 1. In this analysis, NaHCO_3_, inoculum size and pH were chosen as independent variables and the carbon dioxide (CO_2_) removal rate (%) and its corresponding chlorophyll content were taken as dependent output response variable.

**Table 2:** Central composite design matrix with coded and uncoded variables

Run	*A*	*B*	*C*	pH	NaHCO_3_ (g/l)	Inoculum size (%)
1	−1	−1	−1	4.0	3.33	10
2	1	−1	−1	8.0	3.33	10
3	−1	1	−1	4.0	10	10
4	1	1	−1	8.0	10	10
5	−1	−1	1	4.0	3.33	30
6	1	−1	1	8.0	3.33	30
7	−1	1	1	4.0	10	30
8	1	1	1	8.0	10	30
9	−1.68179	0	0	2.6	6.66	20
10	1.68179	0	0	9.4	6.66	20
11	0	−1.68179	0	6.0	1.06	20
12	0	1.68179	0	6.0	12.27	20
13	0	0	−1.68179	6.0	6.66	3.18
14	0	0	1.68179	6.0	6.66	36.81
15	0	0	0	6.0	6.66	20
16	0	0	0	6.0	6.66	20
17	0	0	0	6.0	6.66	20
18	0	0	0	6.0	6.66	20
19	0	0	0	6.0	6.66	20
20	0	0	0	6.0	6.66	20

The three independent variables were varied over two levels with pH between (4 and 8) relative to the centre point (pH 6), the second independent variable (NaHCO_3_ in g/l) was varied over two levels (3.3 and 9.9 g/l) relative to the centre point (6.6 g/l) and the third independent variable (inoculum size in %) was varied over two levels (10 and 30 %) relative to the centre point (20 %).

The full factorial CCD matrices of three variables with respect to their uncoded (real) and coded values are presented in Table 2. The response surface method was constructed using MINITAB 16 statistical software. Evaluation of the goodness of fit of the model is done through coefficient determination and analysis of variances. The experimental results were fitted to a second order polynomial Eq. (1):

1 $$Y = \beta_{0} + \beta_{1} A + \beta_{2} B + \beta_{3} C + \beta_{11} A^{2} + \beta_{22} B^{2} + \beta_{33} C^{2} + \beta_{12} AB + \beta_{13} AC + \beta_{23} BC$$

where *Y* is the dependent variable (CO_2_ removal and chlorophyll content); *A*, *B* and *C* are the independent variable; *β*_0_ is the regression coefficient at center point; *β*_1_, *β*_2_ and *β*_3_ are the linear coefficients; *β*_11_, *β*_22_ and *β*_33_ are the quadratic coefficients and *β*_12_, *β*_13_ and *β*_23_ are the second-order interaction coefficients. The developed regression model was evaluated by analyzing the values of regression coefficients, analysis of variance (ANOVA), *P* and *F* values. The quality of fit of the polynomial model equation was expressed by the coefficient of determination, *R* ^2^. The statistical software package was used to identify the experimental design as well as to generate a regression model to predict the optimum combinations considering the effects of linear, quadratic and interactive effects on CO_2_ removal and corresponding chlorophyll content.

### Carbon dioxide removal

Alkalinity based titrimetric method was used to find out the dissolved inorganic carbon species (${\text{H}}_{2} {\text{CO}}_{3}^{*}$, HCO_3_^−^, CO_3_^2−^) by following standard method (APHA 2005). Briefly, all the samples were centrifuged to obtain supernatant which was titrated against standardized H_2_SO_4_ (0.02 N). In a typical experiment, 10 ml of supernatant was taken in a beaker and titrated against H_2_SO_4_ using phenolphthalein (p*K*a 8.6) indicator. After the first end point, titration was continued using methyl orange (p*K*a 3.8) as the indicator to get the second end point. The readings were noted down and the dissolved inorganic carbon species (${\text{H}}_{2} {\text{CO}}_{3}^{*}$, HCO_3_^−^, CO_3_^2−^) were determined using the formulae in standard method and the sum of the three species are represented as total inorganic carbon species CO_2_ as mg CaCO_3_/l which is shown in Eq. (2). Furthermore, the ratio of CO_2_ to CaCO_3_ is 1.4 which is taken for CO_2_ estimation from total inorganic carbon species and the same has been shown in the Eq. (3) (Kemmer 1979).

2 $${\text{CO}}_{{2({\text{asCaCO}}_{3} )}} {\text{ mg}}/{\text{l}} = {\text{H}}_{2} {\text{CO}}_{3}^{*} + {\text{HCO}}_{3} + {\text{CO}}_{3}^{2 - }$$

3 $${\text{CO}}_{{2({\text{asCaCO}}_{3} )}} {\text{ mg}}/{\text{l}} \div 1.14 = {\text{CO}}_{{2({\text{as}}{\text{CO}}_{2} )}} {\text{ mg}}/{\text{l}}$$

The CO_2_ removal (%) was determined by calculating difference between the initial concentration of CO_2_ (based on Eq. 3) from each experimental runs and final concentration after growth in stationery phase (until no observation of CO_2_ removal was found) and is expressed below

4 $${\text{CO}}_{2} \,{\text{removal}}\,(\% ) = \frac{{{\text{Initial}}\,{\text{CO}}_{2} - {\text{final}}\,{\text{CO}}_{2} }}{{{\text{Initial}}\,{\text{CO}}_{2} }} \times 100$$

### Chlorophyll analysis

The chlorophyll content in the medium is determined by spectrometric analysis. Briefly, 5 ml algae culture was centrifuged at 10,000 rpm for 10 min. The supernatant was drained off and the sample was re-suspended in ethanol/diethyl ether and kept boiling for 5 min. After boiling, the sample was made up to 5 ml with ethanol/diethyl ether. The optical density was measured at 660 nm and 642.5 nm with solvent as a blank. The chlorophyll content was determined using the formula (Becker 1994):

5 $${\text{Chlorophyll}}\,\left( {{\text{mg}}/{\text{l}}} \right) = \left( {9.9*{\text{OD}}_{660} } \right) - \left( {0.77*{\text{OD}}_{642.5} } \right)$$

## Results and discussion

### Central composite design: response surface estimation

**Fig. 2:**
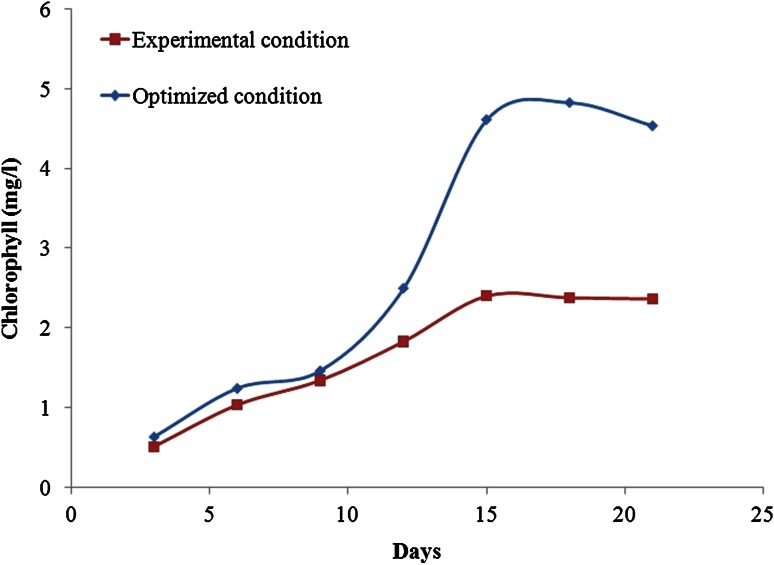
Growth profile of microalgae under optimized condition [pH 8, NaHCO_3_ 3.33 (g/l), inoculum size −30 (%)] and experimental condition

The growth of *C. pyrenoidosa* under experimental conditions and optimized condition is shown in Fig. 2. It can be noted that, there was a twofold increase in the chlorophyll accumulation in the optimized condition compared to the experimental condition which may be due to the availability of carbon source that helps in synthesizing chlorophyll. The experiments based on design (Table 2) were lasted for 20 days after which the growth was stationery and hence no further improvement was observed on chlorophyll accumulation and CO_2_ removal and the same has been given as experimental data in RSM. The results of CO_2_ removal and chlorophyll content (both predicted and experimental) for different design variables (pH, inoculum size and NaHCO_3_) are presented in Table 3. The second-order polynomial Eq. (1) are fitted with experimental results of maximum CO_2_ removal (%) and corresponding chlorophyll content from the estimated regression coefficients. The regression coefficients for CO_2_ removal and chlorophyll content are presented as Eqs. (6) and (7), respectively.

6 $$Y = 67.6120 + 9.8638A - 6.6875B + 8.3419C + 0.0318A^{2} - 0.3245B^{2} - 1.5856C^{2} + 2.9458AB - 5.0963AC - 0.6830BC$$

7 $$Y = 2.9031 + 0.6274A - 0.2143B + 0.1584C - 0.1050A^{2} + 0.3559B^{2} + 0.7672C^{2} - 0.3266AB - 0.8803AC - 0.7276BC$$

**Table 3:** Central composite design matrix and the output responses for CO_2_ removal and chlorophyll

Run	pH	NaHCO_3_ (g/l)	Inoculum size (%)	CO_2_ removal (%)_(experimental)_	CO_2_ removal (%)_(predicted)_	Chlorophyll (mg/l)_(experimental)_	Chlorophyll (mg/l)_(predicted)_
1	4.0	3.33	10	53.07	51.49	1.016	1.415
2	8.0	3.33	10	80.00	75.52	5.240	5.083
3	4.0	10.00	10	33.95	33.39	3.499	3.095
4	8.0	10.00	10	74.37	69.20	5.064	5.457
5	4.0	3.33	30	78.07	79.73	5.263	4.947
6	8.0	3.33	30	86.32	83.38	4.614	5.095
7	4.0	10.00	30	57.93	58.90	3.484	3.717
8	8.0	10.00	30	76.25	74.33	2.880	2.558
9	2.6	6.66	20	53.07	51.11	1.461	1.550
10	9.4	6.66	20	77.37	84.29	3.858	3.661
11	6.0	1.06	20	75.42	78.07	4.475	4.270
12	6.0	12.27	20	52.98	55.25	3.452	3.549
13	6.0	6.66	3.18	43.75	49.07	4.906	4.806
14	6.0	6.66	36.81	77.50	77.13	5.347	5.339
15	6.0	6.66	20	67.10	66.96	2.895	2.898
16	6.0	6.66	20	67.10	66.96	2.895	2.898
17	6.0	6.66	20	67.10	66.96	2.895	2.898
18	6.0	6.66	20	67.10	66.96	2.895	2.898
19	6.0	6.66	20	67.10	66.96	2.895	2.898
20	6.0	6.66	20	67.10	66.96	2.895	2.898

### Analysis of variance and residuals

The regression helps to correlate the experimental data with predicted response. *R* ^2^ and adjusted *R* ^2^ values represent the proportion of variation in the response that is explained by the model where *R* describes the amount of variation in the observed responses. The value of *R* ^2^ is also a measure of fit of the model and the adjusted *R* ^2^ value compares models with different independent variables. The ANOVA results are presented in Tables 4 and 5 and it can be noted that, the *R* ^2^ values for CO_2_ removal and corresponding chlorophyll content are 0.9527 and 0.962 which indicates high degree of correlation between experimental and predicted values.

**Table 4:** ANOVA for fit of CO_2_ removal (%) from central composite design

Sources of variation	Sum of squares	Degree of freedom	Mean square	*F* value	*P*
Regression	3266.42	9	358.49	22.37	0.0000
Residuals	159.50	10	16.02		
Total	3386.65				

*R*_(pred.)_^2^ = 95.27 %; *R*_(adj.)_^2^ = 91.01 %

**Table 5:** ANOVA for fit of chlorophyll estimation from central composite design

Sources of variation	Sum of squares	Degrees of freedom	Mean square	*F* value	*P*
Regression	27.8792	9	3.09769	28.24	0.000
Residuals	1.0999	10	0.10999		
Total	28.9791				

*R*_(pred.)_^2^ = 96.20 %; *R*_(adj.)_^2^ = 92.79 %

The estimated regression coefficients for removal of CO_2_ and chlorophyll content are presented in Tables 6 and 7, respectively, along with their corresponding *P* value and *T* values. It can be observed from Table 6 for CO_2_ removal (%) that, the coefficient for single effect of pH (*β*_1_), sodium bicarbonate (*β*_2_) and inoculum size (*β*_3_) (*P* < 0.050) are highly significant whereas the square effects i.e., linear co-efficient *β*_11_, *β*_22_ and *β*_33_ and the interactive terms *β*_23_ are not significant except *β*_13_ which is significant. From Table 7 (chlorophyll content) the coefficients for single effect except inoculum size (*β*_3_) are significant whereas the interactive and square effects except *β*_11_ are significant.

**Table 6:** Estimated regression coefficients for CO_2_ removal (%)

Term	Coefficient	Standard error	*T*	*P*
*β*_0_	67.6120	1.6323	41.415	0.000
*β*_1_	9.8638	1.083	9.107	0.000
*β*_2_	−6.7875	1.083	−6.266	0.000
*β*_3_	8.3419	1.083	7.701	0.000
*β*_11_	0.0318	1.054	0.030	0.977
*β*_22_	−0.3245	1.054	−0.308	0.765
*β*_33_	−1.5856	1.054	−1.504	0.164
*β*_12_	2.9458	1.415	2.081	0.064
*β*_13_	−5.0963	1.415	−3.601	0.005
*β*_23_	−0.6830	1.415	−0.483	0.640

**Table 7:** Estimated regression coefficients for chlorophyll

Term	Coefficient	Standard error	*T*	*P*
*β*_0_	2.9031	0.13526	21.463	0.000
*β*_1_	0.6274	0.08974	6.991	0.000
*β*_2_	−0.2143	0.08974	−2.388	0.038
*β*_3_	0.1584	0.08974	1.765	0.108
*β*_11_	−0.1050	0.08736	−1.202	0.257
*β*_22_	0.3559	0.08736	4.074	0.002
*β*_33_	0.7672	0.08736	8.781	0.000
*β*_12_	−0.3266	0.11726	−2.786	0.019
*β*_13_	−0.8803	0.11726	−7.513	0.000
*β*_23_	−0.7276	0.11726	−6.210	0.000

### Response surface plots for CO_2_ removal

**Fig. 3:**
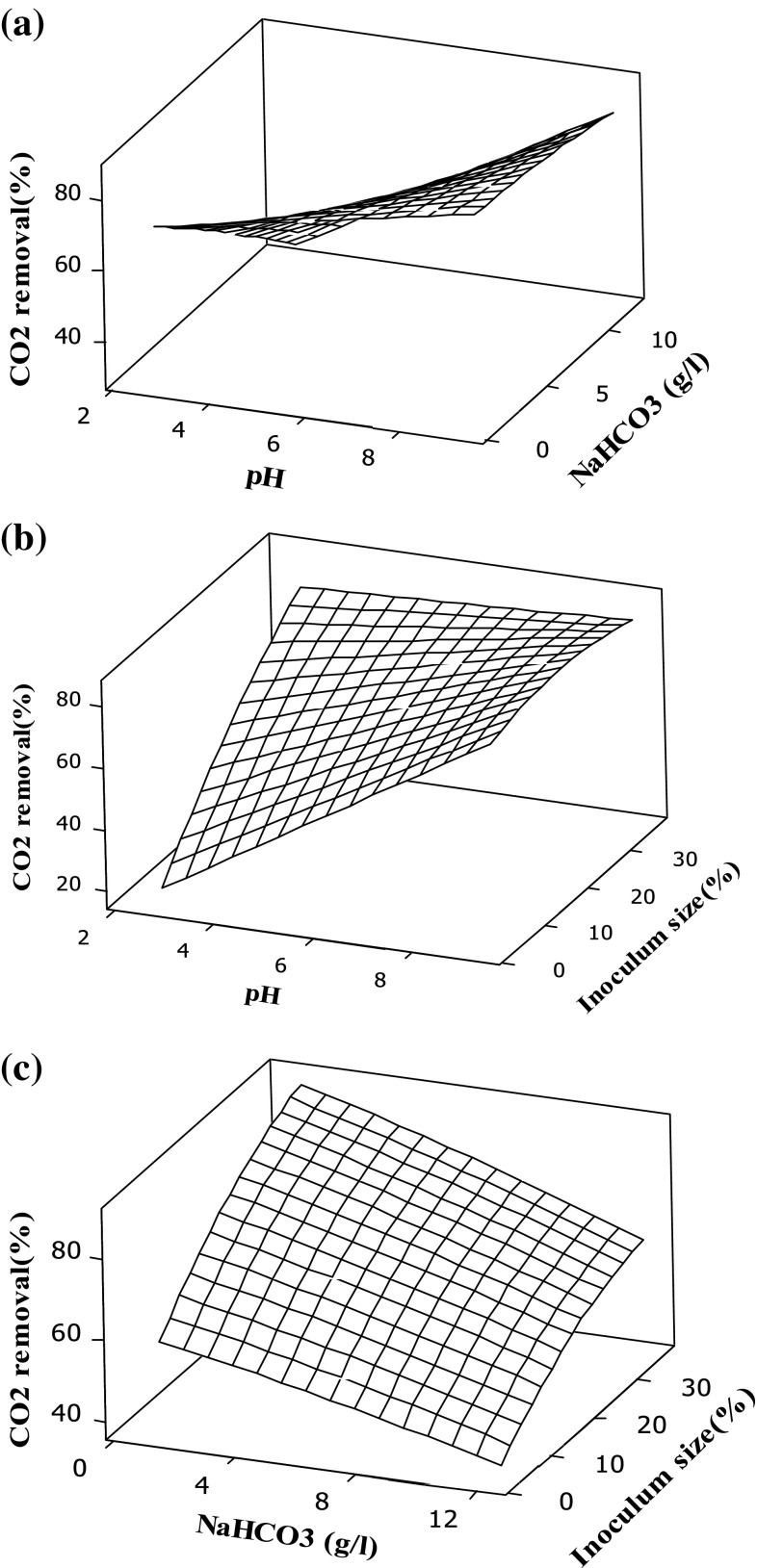
Surface plot for CO_2_ removal (%). **a** Conc. of NaHCO_3_ (g/l), pH. **b** Inoculum size (%), pH. **c** Inoculum size (%), Conc. of NaHCO_3_ (g/l)

The main objective of the response surface method is to find out the optimum condition for maximum CO_2_ removal with respect to the chosen variables. The interaction effects between the variables (pH and sodium bicarbonate) for carbon dioxide removal is presented in Fig. 3a, as the sodium bicarbonate concentration increases concomitantly with increase in pH, the removal of CO_2_ increases. This is due to the fact that the microalgae uptake the HCO_3_ inside the cell and by the action of carbonic anhydrase, it converts HCO_3_ into CO_2_ inside the cell. The observations are consistent with the results reported in the literature (Devgoswami et al. 2011).

Similarly, the interaction effects between the variables (inoculum size and pH) for carbon dioxide removal is presented in Fig. 3b, it can be observed at low pH, even if the inoculum size is high; there is a decrease in the CO_2_ removal. This could be due to the fact that at low pH all the carbon dioxide will exist in the form of free CO_2_ (H_2_CO_3_) as the algae cannot utilize for metabolic activity (Van Den Hende et al. 2012). Figure 3c depicts the interaction effect between the variables (inoculum size and sodium bicarbonate) for CO_2_ removal. It can be noted from the figure that, increase in inoculum size with concomitant decrease in sodium bicarbonate concentration, helps to increase the uptake of CO_2_ (i.e. CO_2_ removal). This could be due to the regulation of H^+^ ions into the cell takes place due to which the hydroxide is formed which in turn affects the CO_2_ removal process (Yeh et al. 2010). In addition, it is also reported that maximum CO_2_ removal varies with different environment conditions. It is reported that at pH 4 and high bicarbonate concentration (0.3 g/l), maximum removal of CO_2_ (82.5 %) was achieved (Lam and Lee 2013). Similarly, in another study (Yeh and Chang 2012), it is reported that a maximum removal of CO_2_ in the range of 90–100 % was achieved with increasing bicarbonate concentration (till 1600 mg/l).

In the present study, the maximum absorption of CO_2_ was achieved at pH 8 with initial NaHCO_3_ concentration of 3.33 g/l and higher inoculum size 30 %. However, with increase in concentration of bicarbonate, there is no significant change in CO_2_ removal which is due to the fact that additional carbon mechanism results in decreased growth rate causing them in reduction of fixing CO_2_ rates (Lam and Lee 2013).

### Response surface plots for chlorophyll content

Chlorophyll *a* is the photosynthetic pigment that is widely present in all eukaryotic microalgae and is synthesized during photosynthetic process as mentioned in Eq. (8). Initially the inorganic carbon is accumulated into microalgal cell by capture of light energy by pigments such as chlorophyll *a* and *b* that are presented in chloroplastida of cell (Wang et al. 2012).

8 $$6{\text{CO}}_{2} + 6{\text{H}}_{2} {\text{O}} + h\nu \left( {\text{sunlight}} \right) \to {\text{C}}_{6} {\text{H}}_{12} {\text{O}}_{6}$$

**Fig. 4:**
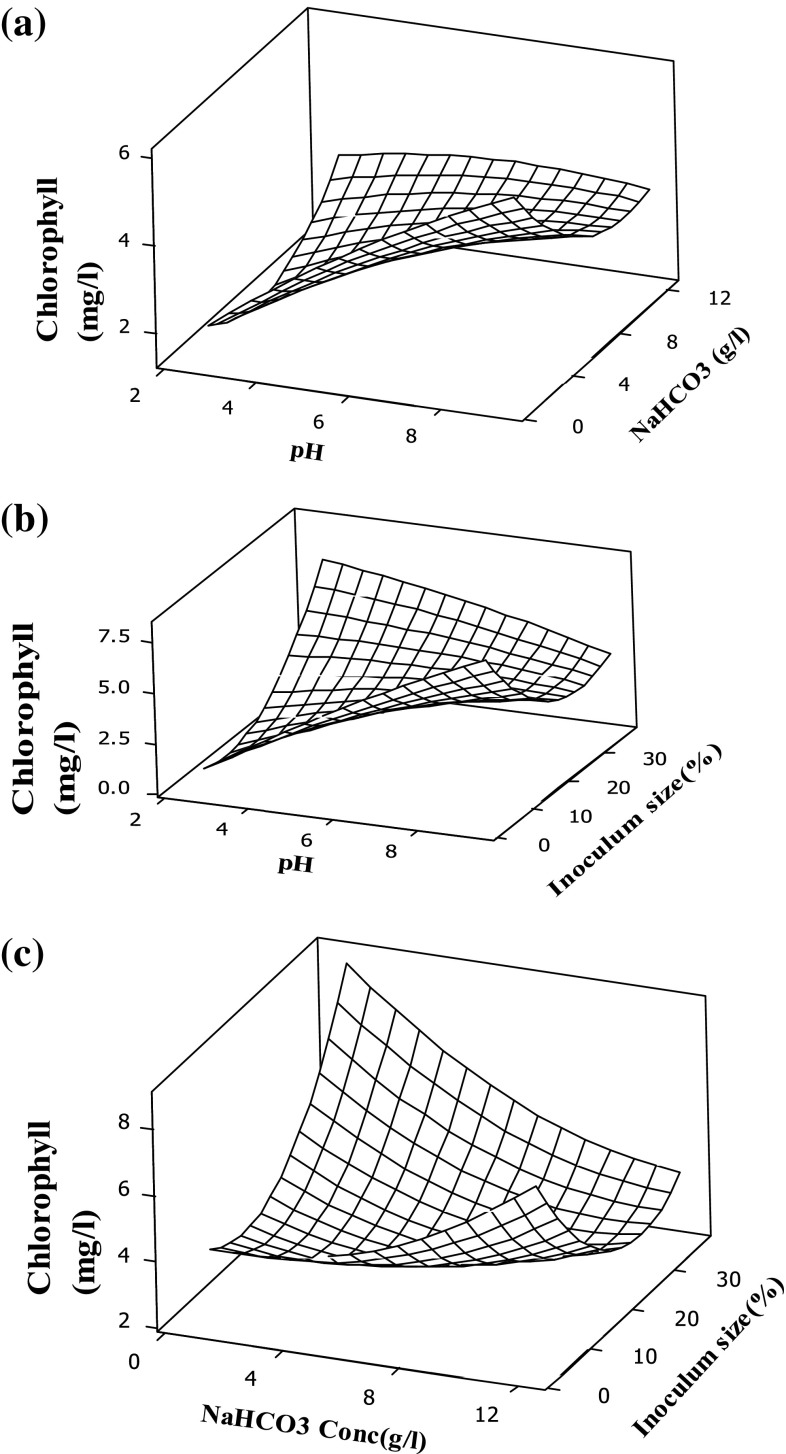
Surface plot for chlorophyll (mg/l). **a** Conc. of NaHCO_3_ (g/l), pH. **b** Inoculum size (%), pH. **c** Inoculum size (%), Conc. of NaHCO_3_ (g/l)

The initial concentrations of chlorophyll are 0.5, 1, 1.34 mg/l for inoculum sizes 10, 20, 30 %, respectively. The surface plots for the interaction effects between the variables (pH and sodium bicarbonate) for the chlorophyll content are presented in Fig. 4a. It can be observed from the figure that, at low sodium bicarbonate concentration, as the pH increases the chlorophyll content increases drastically but at higher sodium bicarbonate concentration only minor variations of chlorophyll content was observed in all pH. This could be due to the excess HCO_3_^−^ (Ci) ions which are not utilized by microalgae due to insufficient light, as synthesis of chlorophyll will mainly depend upon the light energy during photosynthesis thereby hindrance the process of synthesizing chlorophyll (Amoroso et al. 1998).

Figure 4b depicts the interaction effects between the variables (inoculum size and pH) for chlorophyll and it can be noticed that at low inoculum size, the chlorophyll content increases with increase in pH. However, the chlorophyll content decreases when the inoculum size increases along with increase in pH. It may be due to the fact that increase in inoculum size causes reduction of H^+^ ions form the medium (Liu et al. 2014; Chi et al. 2011).

The interaction effects between the variables (inoculum size and sodium bicarbonate) are presented in Fig. 4c and it can be observed from the figure, that the chlorophyll content decreases when the sodium bicarbonate increases along with the increase in inoculum size. This is due to the fact that the increase in sodium bicarbonate decreases the photosynthetic efficiency. The obtained results are in consistent with the results reported in the literature (White et al. 2013).

At higher concentration of bicarbonate and pH, there is no significant change in chlorophyll content which could be due to: (1) increasing rates of photorespiration (2) availability of C:N ratio in the medium as the nitrogen is also one of the factor for chlorophyll assimilation.

## Conclusion

The interactive effects between the variables (pH, inoculum size and sodium bicarbonate concentration) for CO_2_ removal using *C. pyrenoidosa* in synthetic medium under mixotrophic condition was investigated by employing response surface method with CCD. The maximum removal of CO_2_ (86 %) was achieved at pH 8 with sodium bicarbonate concentration of 3.33 g/l, and inoculum size of 30 %. The regression value of 0.9527 and 0.962 was observed for CO_2_ removal and chlorophyll content which implies that the experimental results are statistically significant. The results reveal that *C. pyrenoidosa* can be used effectively for bio-fixation of CO_2_ in the form of bicarbonate at alkaline conditions in higher inoculum size under mixotrophic condition. Further investigations on adapting microalgae at alkaline conditions will be a useful for large-scale applications to develop bioenergy feedstock.
