# Erratum to: Evaluation of photosynthetic efficacy and CO_2_ removal of microalgae grown in an enriched bicarbonate medium

**DOI:** 10.1007/s13205-016-0374-1

**Published:** 2016-02-17

**Authors:** S. Abinandan, S. Shanthakumar

**Affiliations:** Environmental Engineering Division, School of Mechanical and Building Sciences, VIT University, Vellore, 632014 India

## Erratum to: 3 Biotech (2016) 6:9 DOI 10.1007/s13205-015-0314-5

Inadvertently, the Fig. 1 was published incorrectly in the original publication. The correct Fig. 1 is given in the following page. The original article has been updated accordingly.

**Fig. 1:**
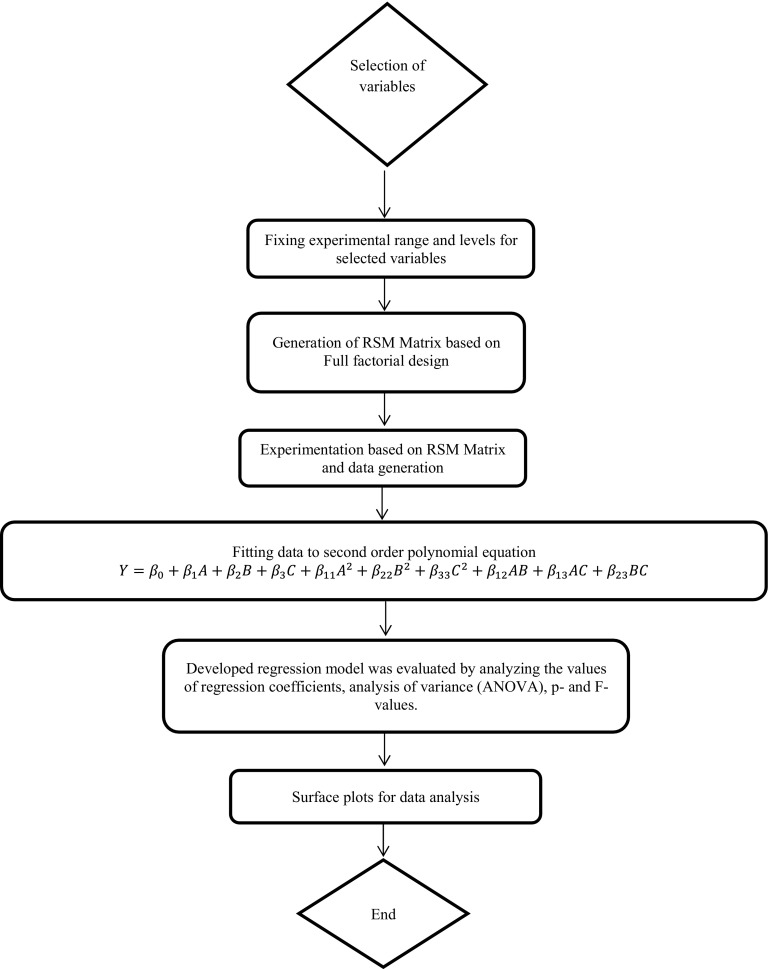
Flow chart representation of response surface methodology

